# Role of Bioactive Compounds in Neuroprotection and Neurodegenerative Disease

**DOI:** 10.3390/nu17193069

**Published:** 2025-09-26

**Authors:** Irene Cantarero, Carmen del Río

**Affiliations:** 1 Departamento de Ciencias Morfológicas y Sociosanitarias, Facultad de Medicina y Enfermería, Universidad de Córdoba, 14004 Córdoba, Spain; 2 Instituto Maimónides de Investigación Biomédica de Córdoba (IMIBIC), Hospital Universitario Reina Sofía, Universidad de Córdoba, 14004 Córdoba, Spain; 3 Instituto de Biomedicina de Sevilla (IBiS), Hospital Universitario Virgen del Rocío (CSIC), Universidad de Sevilla, 41013 Sevilla, Spain; crio-ibis@us.es; 4 Departamento de Biología Celular, Facultad de Biología, Universidad de Sevilla, 41013 Sevilla, Spain

The prevalence of neurological disorders, including neurodegenerative conditions such as Alzheimer’s and Parkinson’s disease and acute conditions like stroke, is increasing due to population aging. As a result, the current burden of these disorders on public health is increasing. They are characterized by progressive neuronal loss, cognitive and functional decline, and a significant reduction in quality of life. Current pharmacological treatments for neurological diseases offer only partial symptom relief and fail to adequately address the underlying multifactorial pathophysiology, including oxidative stress, neuroinflammation, mitochondrial dysfunction, cholinergic system impairment, neurovascular damage, and disrupted neurotrophic signaling [1].

In recent years, natural product-based interventions, including phytochemical formulations and microbiota-derived metabolites, have emerged as promising, multi-target strategies for neuroprotection. This Special Issue aims to synthesize findings from recent studies that highlight the diversity of approaches and the translational potential of bioactive compounds in promoting brain health and resilience against neurological diseases.

The search for effective neuroprotective agents increasingly points toward natural products and their bioactive constituents. A key aspect is the optimization of extraction processes, as elegantly demonstrated with *Ferula persica*, where green pressurized liquid extraction enhanced the yield of diverse compounds that possessed antioxidant, anti-inflammatory, and anticholinesterase properties. Such methodological advances are essential to fully explore the neuroprotective potential of complex plant matrices [2].

Neuroinflammation remains a central mechanism in brain disorders. In this sense, a study on *Areca catechu* extract and its constituent (−)-epicatechin demonstrated suppression of proinflammatory cytokine release and oxidative stress in microglial cells through MAPK and NF-κB modulation. Such findings provide in vitro support for their potential as anti-neuroinflammatory agents [3]. Moving on to preclinical evidence, a JP1 formulation combining silkworm hydrolysate with ginger and holy basil extracts in black glutinous rice-derived film proved effective in models of stress and stroke by reducing oxidative damage, apoptosis, and stress hormone levels while improving cholinergic function [4]. The translational trajectory of bioactive compounds was further extended in a clinical study, where *Salicornia ramosissima* extract was administered to patients after transient ischemic attack and minor stroke, reducing blood pressure and homocysteine levels as a vascular damage marker, and showing a slight effect on cognitive performance, suggesting a role in recovery and secondary stroke prevention [5].

Flavonoids have demonstrated remarkable potential as natural inhibitors of the enzyme acetylcholinesterase (AChE), according to various preliminary studies conducted both in vitro and through in silico approaches. These bioactive compounds, abundantly present in fruits, vegetables, and medicinal plants, not only target AChE but also exert broader neuroprotective effects by modulating cholinergic neurotransmission. A review published in this Special Issue specifically addresses the mechanistic and therapeutic insights into flavonoid-based AChE inhibition, highlighting their relevance for neurodegenerative diseases. Given the pivotal role of acetylcholine in memory and learning, this inhibition is particularly significant, as it may help preserve synaptic function and mitigate cognitive decline [6,7].

In addition to these plant-derived compounds, their gut microbiota-generated metabolites are also being investigated. Urolithin A, a metabolite of ellagitannins and ellagic acid, exhibited pronounced neurotrophic and antidepressant-like effects in both cellular and animal models. By activating the AMPK/CREB/BDNF signaling cascade, it mitigated stress-induced neuronal damage and neuroinflammation, underscoring the therapeutic potential of gut-derived metabolites for supporting brain health [8].

Finally, a review on bioactive substances in different mushrooms species broadens the scope of this topic by emphasizing their long-recognized but underexplored potential as functional foods for mental health. While clinical validation is still limited, accumulating preclinical evidence suggests that mushroom-derived nutraceuticals could contribute to holistic dietary strategies supporting brain health [9].

Therefore, this Special Issue brings together a compelling body of work that bridges molecular insights with translational promise. By highlighting innovative interventions, from urolithin A to mushroom-based nutrients and synergistic blends, the collection advances our understanding of how nutrition and bioactives contribute to brain health and recovery. We hope these findings catalyze further interdisciplinary exploration, encouraging collaborations that span discovery science, clinical application, and public health impact.
